# Do human–wildlife interactions predict offspring hiding strategies in peri-urban fallow deer?

**DOI:** 10.1098/rsos.231470

**Published:** 2024-03-20

**Authors:** Jane Faull, Kimberly Conteddu, Laura L. Griffin, Bawan Amin, Adam F. Smith, Amy Haigh, Simone Ciuti

**Affiliations:** ^1^ Laboratory of Wildlife Ecology and Behaviour, SBES, University College Dublin, Dublin 4, Ireland; ^2^ Department of Forest Resources Management, University of British Columbia, 2424 Main, Mall, Vancouver V6T 1Z4, Canada; ^3^ The Frankfurt Zoological Society, Frankfurt, Germany; ^4^ Faculty of Environment and Natural Resources, Department of Wildlife Ecology and Management, University of Freiburg, Freiburg, Germany

**Keywords:** artificial feeding, anti-predator strategies, bedsite selection, human–wildlife coexistence, maternal behaviour, human–wildlife feeding interactions

## Abstract

Human activities can induce significant behavioural changes in wildlife. Often explored through extractive interactions (e.g. hunting) that can favour certain behavioural traits, the implications of non-extractive ones, such as wildlife feeding, remain understudied. Research shows that people tend to favour bolder individuals within populations despite their dynamics and consequences being unclear. Using fallow deer in a peri-urban environment, we studied whether mothers that show reduced fear of humans and consistently approach them for food adopt weaker anti-predator strategies by selecting less concealed fawning bedsites closer to human hotspots. This would provide the advantage of additional feeding opportunities in comparison with shyer mothers while keeping their fawns close. Our dataset encompassed 281 capture events of 172 fawns from 110 mothers across 4 years. Surprisingly, mothers that regularly accepted food from humans selected more concealed bedsites farther from human hotspots, giving their offspring better protection while also benefitting from additional food during lactation. Our results show behavioural adaptations by a subset of females and, for the first time, link the tendency to approach humans and strategies to protect offspring. Given previous findings that these begging females also deliver heavier fawns at birth, our research further investigates human–wildlife feeding interactions and their behavioural implications.

## 1. Introduction

As humans and wildlife coexist and interact in all walks of life, we have influenced wildlife behaviour in countless ways. Hunting and fishing have been shown to promote the selection of both behavioural and morphological traits in wildlife [1–3]. However, humans can intrude upon wildlife habitats and manipulate their behaviour in several additional ways. Wildlife species must contend with increases in habitat disturbance or loss, scattered or fragmented food sources, changes in ecosystem functioning, frequent human encounters and the emergence of novel communities. In addition, conflicts arise as they move into towns and cities or these urban areas expand and invade their habitats [4,5]. To successfully live in human-dominated landscapes, many animals have adopted novel behaviours that allow them to use readily available anthropogenically sourced resources [5–7]. How behavioural traits can drive the adaptability of wild animals to human-dominated landscapes and how these traits affect the conservation status of species remains largely unknown [8]. Despite these knowledge gaps, there are several examples in the literature of behavioural adaptations that wildlife have developed to exploit human environments. Gull species, including lesser black-backed gull (*Larus fuscus*) and herring gull (*Larus argentatus*), alter their use of foraging patches throughout the day in response to human activity patterns [9]. The Australian white ibis (*Threskiornis molucca*) is known to scavenge through anthropogenic waste, causing local management problems due to the spread of pathogens [10]. Other species have begun using human-derived food sources more directly through artificial feeding activities. For instance, in response to climate change and garden feeding, the Eurasian blackcap (*Sylvia atricapilla*) has altered its winter migratory behaviour and now overwinters in Northern Europe [11].

Human–wildlife interactions can be classified as extractive, where resources are removed from the ecosystem by humans, or non-extractive, where interactions occur but nothing is taken away [12]. Extractive interactions like hunting and fishing involve the removal of animals from the ecosystem, and the impacts of these activities have been extensively investigated [13,14]. Artificial feeding is classified as a non-extractive interaction and can fall into two categories: accidental and intentional [15]. Accidental feeding occurs when animals feed on anthropogenic food sources that were not intended for feeding. For example, gulls eat food waste left behind in green areas and parks [16]. Free-ranging dogs (*Canis familiaris*) in less developed countries are known for scavenging human waste [17]. In contrast, intentional feeding occurs when food is provided with the distinct goal of feeding animals. Feeding wildlife has grown in popularity probably because it allows for close interactions. Despite becoming more common, it is strongly discouraged due to links between wildlife feeding and disease transmission, increased aggression and personal injury [18,19]. Most commonly, we see examples of people feeding garden birds [20], terrestrial mammals [21–23] and marine megafauna [24,25]. Human perception of wildlife has particularly changed within urban and peri-urban settings. People have started to interact with and provide food to birds and mammals and this has been increasing steadily across the globe [12], with unknown ultimate effects on population dynamics and inter-species interactions.

While the public perception may be that feeding wildlife is beneficial for animals, research is now focusing on exploring the potentially harmful and unseen impacts [7,18,19]. The implications of these activities are twofold, with consequences for both humans and wildlife [12]. Considering that the health and fitness of an individual are heavily linked, it is important that we understand how being fed by people impacts the well-being and physiology of an individual [26]. The assumption that the increased availability of food leads to improved body condition and overall better health is often untrue [27–29]. Adult barbary macaques (*Macaca sylvanus*) that are fed by humans exhibit larger body sizes but poorer quality coat conditions, possibly due to a diet insufficient in nutrients provided by tourists [27]. Calves of wild dolphins (*Tursiops* sp.) that engage in tourist feeding experience higher mortality than their non-feeding conspecifics, potentially due to diseases resulting from human contact or pollution [29]. It has also been shown that human presence can elicit a fear response in ungulates similar to (or greater than) that seen for natural non-human predators [30], meaning that individuals fed in close contact could experience increased stress around humans. Associations with individuals who have become conditioned to take food from humans have been shown to increase the likelihood of new individuals participating in feeding interactions [31].

Despite our understanding of the impacts on individuals who engage with humans [7,32,33], it remains unclear how these associations affect future generations. Examining the effects of human–wildlife interactions on parental care and offspring fitness can provide insight into future impacts on offspring if these activities continue and help to inform management and mitigation strategies. Urban and national parklands are popular tourist sites and many provide ample opportunities for hand-feeding and close interaction between visitors and wildlife [12]. The Phoenix Park, situated within the Dublin metropolitan area in Ireland, is Europe’s largest enclosed urban park. Based on the context provided, we investigated anti-predator strategies adopted by fallow deer (*Dama dama*) females during weaning in a population living in this peri-urban area where human–wildlife feeding interactions occur. Despite being strongly discouraged by management, feeding the deer has become commonplace in the park [7,32]. This popular activity has led researchers to identify a spectrum of distinct behavioural types in the population to date, consisting of acceptors who show reduced fear responses to humans and avoiders [7]. Acceptors often boldly approach humans begging for and accepting food (known as consistent beggars hereafter) and account for approximately 20% of the population, which tolerates close contact with humans and associates them with food [7]. In comparison, shyer individuals have a lower tolerance for human presence, either avoiding engagement with humans attempting to interact or actively moving away from groups of people [7].

Our research specifically aims to investigate the anti-predator strategies of mothers during the fawning season as a function of their willingness to accept food from humans. In our study site, on the one hand, park visitors tend to feed the deer willing to accept food [7], and on the other hand, deer of all sex and age classes are targeted by annual culls to maintain this population at sustainable levels, and fawns are seldom preyed upon by unleashed dogs of park visitors [34]. Therefore, females in this population must balance their fear of humans [30,35] with their willingness to approach them to obtain extra food and the need to hide their fawns far from humans and their dogs. Our main prediction is that females that are less afraid of humans and regularly accept food from them may have compromised their anti-predator strategies during weaning. Specifically, we predict that bolder mothers that commonly accept food from park visitors would hide their fawns in bedsites closer to human feeding hotspots, allowing them to access feeding opportunities more easily. Feeding occurs within accessible hotspot open areas of the park, which are far away from forest patches (characterized by dense understorey vegetation less accessible to park visitors), so hiding fawns closer to the hotspots of human feeding may be linked with poorer conditions for fawn concealment. These bedsites may have higher visibility from most directions, leaving them open to discovery by unleashed dogs and the natural predator, the red fox (*Vulpes vulpes*) and potentially more subjected to harsh weather conditions. Furthermore, we predict that the link between a mother’s willingness to accept food from humans and bedsite selection (closer to human hotspots) would be stronger during the first days of a fawn’s life—when the decision of where the neonate fawn would be delivered and concealed is expected to be entirely taken by the mother—and weaker for bedsite locations occupied by older, more mobile and independent fawns [36].

## 2. Material and methods

### 2.1. Study site and population

**Figure 1.**
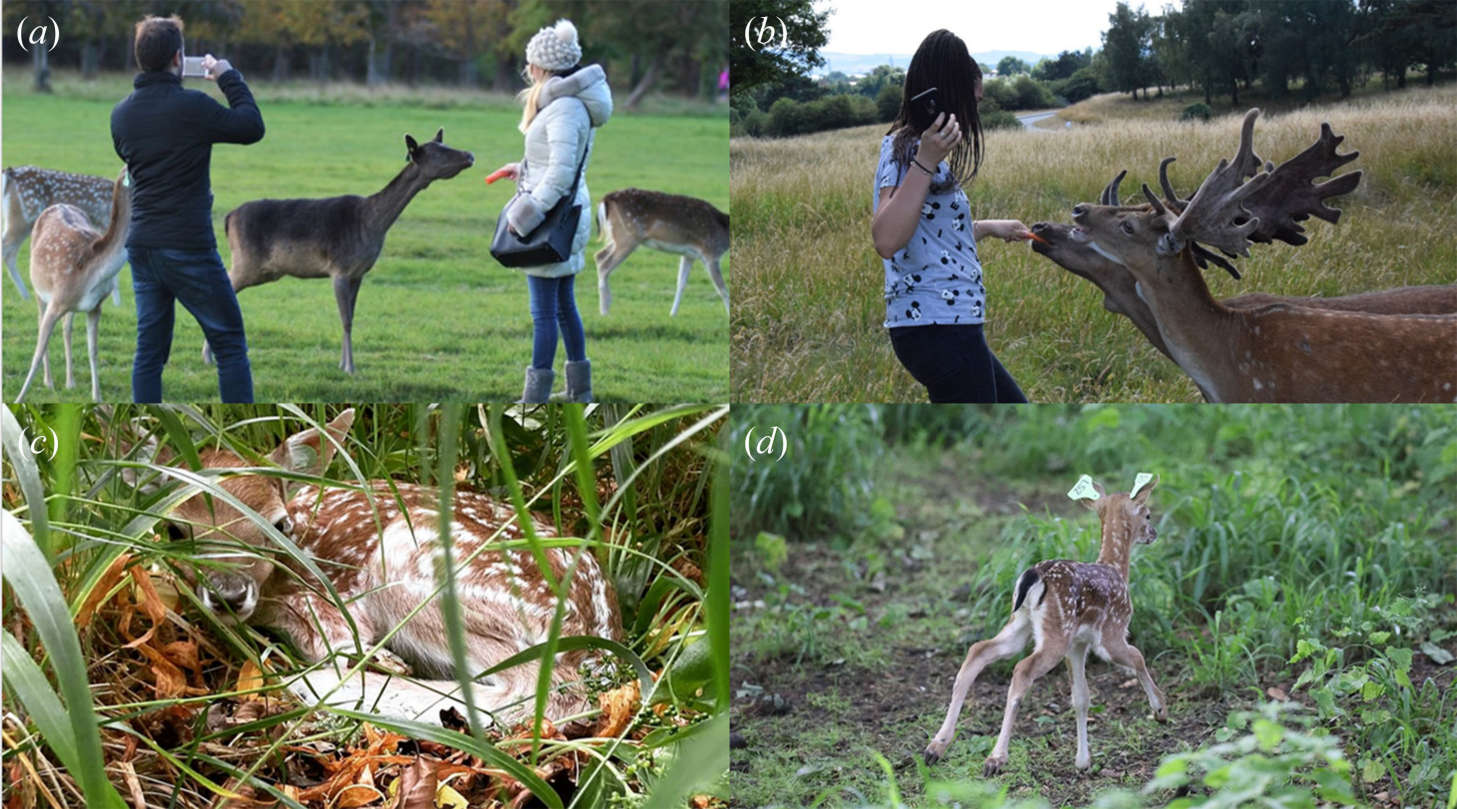
(*a*) Adult females (picture by Laura L. Griffin) and (*b*) adult males (picture by Bawan Amin) being hand-fed with carrots by park visitors; (*c*) neonate fawn hidden in the bedsite vegetation (picture by Clíodhna Hynes); and (*d*) running away at release after being ear-tagged by the capture crew team of University College Dublin (picture by Clíodhna Hynes).

We conducted our study on a population of fallow deer inhabiting the largest enclosed urban park in Europe, the 7 km^2^ Phoenix Park in Dublin City Centre, Ireland (53.3559° N, 6.3298° W), for 4 years (2018–2021). The fallow deer population was maintained at approximately 600 individuals by annual culls (one park ranger plus one professional deer stalker targeting approximately 75 deer per year across multiple age and sex classes to maintain the natural population structure; Office of Public Works (OPW), official data). We routinely monitored the deer shot and the animal welfare during culling operations. The number of deer removed in terms of behavioural types (individuals consistently begging park visitors for food as opposed to individuals avoiding human–deer interactions) reflected the proportion they occur in the population *sensu* Griffin *et al.* [7]. Annual culls are usually carried out for 2–3 days in the period between November and February, with a period of at least three weeks in between two culls to reduce deer disturbance. Other causes of mortality in this population are traffic collisions and occasional predation upon neonate fawns by foxes (*Vulpes vulpes*) and unleashed dogs [34]. Population density is quite high, which is reflected in below-average female productivity for this species, most likely driven by a density-dependent effect [37], where approximately 40% of females are seen with a fawn by the end of the summer in comparison with greater than 80% in similar British systems [38]. This information is relevant in our human–deer feeding interaction study because female deer, as shown by Griffin *et al.* [7], may have an advantage in getting artificial food from park visitors in an environment with relatively limited natural food sources. Phoenix Park is a public site with roads and walkways running throughout, making much of the park accessible to the public and providing ample opportunities for human interaction with the deer (figure 1) [7]. The park receives an estimated 10 million visitors annually (OPW, official data).

### 2.2. Fawn capture protocol and recording the bedsite location

Fallow deer fawns are ear-tagged (unique colour number and colour letter number combinations) annually during the first three weeks of life, resulting in the vast majority (greater than 80%) of the deer being individually recognizable for management and longitudinal research purposes. Ear-tagging during our study was conducted under animal care permit UCD-AREC-E-18-28, which also covers all non-invasive behavioural observations described in our work. We subdivided the area of our study site that is typically selected by deer mothers to give birth and conceal their fawns into seven sectors, all of which were patrolled by a trained capture team supervised by a certified wildlife biologist. Fallow deer are a hider species, meaning their fawns are concealed among vegetation and left unsupervised in bedsites for long periods of time after birth [39,40]. Depending on the number of bedsites discovered in each area, we visited an average of two–three sectors a day, and each sector was patrolled at least once every 3 days. Once a bedsite was discovered, we captured fawns using circular fishing nets (1–1.5 m diameter) with elongated handles (1–1.5 m length). Captured fawns were sexed and ear-tagged. The fawns were weighed in 100 l cloth bags using digital scales (resolution: 0.01 kg—Dario Markenartikelvertrieb) before being released. The complete capture protocol and description of additional data on neonate fawns can be found in Amin *et al*. [41]. In most cases, fawns were recaptured multiple times (from one to four times) over the fawning period [41], meaning that the bedsite locations of fawns of varying ages (from neonate fawns up to three-week-old fawns) were recorded. It is worth noting that traditional fawning sites in the park are usually located far from the main open areas where visitors (human hotspots) spend most of their time in the park. As shown by Griffin *et al*. [7], female fallow deer in the park share the same areas irrespective of whether they are begging park visitors for food or not. Given a group of females, if they are approached by park visitors, only the beggar females would get closer to visitors accepting food, whereas the others would carry on with their activities (e.g. grazing, resting and walking). When it is time to give birth, females leave the main feeding areas and move to the traditional sites that are located in forested areas with dense understorey vegetation, which are significantly less used by visitors [34]. From here, our main hypothesis is that females usually begging park visitors for food would deliver and keep their fawns closer to human hotspots.

We recorded the bedsite coordinates and determined the average bedsite visibility (*sensu* Bongi *et al.* [42] and Amin *et al.* [41]) using a cardboard square the size of a standing fawn (height: 45 cm and width: 36 cm) that was sectioned off into multiple equally proportioned triangles. The square was held vertically perpendicular to the ground at the bedsite location, such that an observer 10 m away could record the proportion of the square that was visible (i.e. how many triangles) at a height of 70 cm from the ground in four cardinal directions. This height was selected as an arbitrary height that would represent the eye height of foxes and medium- to large-sized dogs, the documented predators of fawns in the park [34].

### 2.3. Willingness of female mothers to accept food from humans (aka begging rank)

Concurrent research in the park collected observational data on human−deer interactions and ranked deer along a continuum ranging from deer avoiding any interaction with humans to deer consistently begging (i.e. approaching humans) for food [7,32]. The majority of adults in the population were tagged as fawns so are individually recognizable. Data collection on deer begging was carried out from May to July each year (2018–2021) as this is an important time of the annual biological cycle of females (late gestation, birth and early weaning) [43]. Females were monitored using a stratified sampling design based on the time of the day, day of the week and area of the park, with data collections occurring from dawn to dusk [7]. The whole park area used by the deer was subdivided into sectors and patrolled on a strictly scheduled basis. Once a herd was identified, observation team members (up to three individuals) identified all deer within the group and collected data on human presence and behaviour (e.g. feeding the deer) and on feeding interactions as they occurred. The start and end time of each interaction, the number of people involved, the identity of the deer involved, what they were fed, how they accepted it (from the hand, thrown to the ground or from a human’s mouth) and any other deer–human or deer–deer interactions that occurred simultaneously (e.g. petting, harassing or dominance) were recorded. The complete procedure for collecting begging interactions is outlined in Griffin *et al*. [7]. This includes how the individual deer were assigned a begging rank, i.e. the best linear unbiased predictors (BLUPs) [44], which corresponds to the random intercept value of a generalized linear mixed-effect model fitted to predict the likelihood of a deer to beg for food (ranging from −2.16 to 5.10). This corresponds to the deer’s willingness to approach humans and accept food after taking into account the group size, time of the day, people present, among many other confounding factors included [7].

### 2.4. Mother–fawn pairs

To determine maternal connections, we observed females of this population between July and August after the fawning season. There is no paternal care in this species [45], so fawns will first appear in the female herd with their mothers. As such, only maternal relationships can be determined, and fathers are unknown. During this time, we recorded interactions such as true suckling, following and social grooming between mothers and fawns including recording the ear tags of both individuals involved. True suckling (aka front suckling) occurs when a fawn feeds from its mother in plain sight of the mother (usually from the front or side) so that she is aware this individual is suckling and allows them. True suckling is distinguished in the field from allosuckling [46], where a fawn approaches a female that is not its mother and attempts to suckle usually from behind, between the back legs or at a right angle to the female [47]. Allosuckling events commonly occur when the correct fawn is suckling from its mother and non-offspring fawns also try to feed [47]. This evokes an aggressive reaction in the female, causing her to chase off all fawns except the one suckling in the correct position. Following behaviour is distinguishable when a fawn stays very close to a female and almost mirrors her actions by moving in sync or very closely behind her. Social grooming can occur either from mother to fawn or fawn to mother. We confirmed a mother–fawn pair after three independent sightings (not occurring in the same observation period) involving one or a combination of these interactions [32]. We only included fawns that had a confirmed mother–fawn pairing as our aim was to investigate whether a mother’s begging rank affected her fawn’s bedsite location. As gestation in this species lasts approximately seven–eight months [45], it was possible for mothers to have multiple fawns during our 4-year study period.

### 2.5. Data handling and analysis

We combined the individual willingness of deer mothers to beg and accept food from park visitors (begging rank *sensu* Griffin *et al.* [7]) with data from their respective fawns. Each row of our final dataset corresponded to a unique capture event of a given fawn at a recorded bedsite along with bedsite visibility, distance (in m) to the most popular hotspot of human feeding in the female sector of the park, identity of the fawn, its weight (in kg), sex, mother’s identity and age (years old). The age of the mother was exact because all individuals in this population were tagged as neonates. We classified the human feeding hotspot by selecting a central point within the area with the most feeding observations recorded in the female sector. This point was used to calculate the distance from the hotspot to the bedsites and test our main hypothesis that begging mothers would conceal their fawns closer to humans. Fawn weight (objective measurement) was used as a proxy for age (instead of subjective age estimated in days by fawn handlers) *sensu* Amin *et al.* [41], because the two metrics were highly correlated (*r*_p_ = 0.762). Heavier (i.e. older) fawns are usually more mobile and independent than lighter (i.e. younger) fawns, as also confirmed in the field by a clear positive relationship between the age of the fawns and difficulty capturing them. Body weight is also a good proxy for fawn quality, as shown previously in this population [48].

Multivariate mixed-effect models provide a means to assess whether there is a correlation between multiple behaviours, either exclusively at the among-individual level of variation or at the within-individual level of variation [49]. As a result, their use in behavioural studies is becoming increasingly popular [50–52]. We used multivariate mixed-effect models to estimate the link between mother-begging behaviour and the characteristics of the selected bedsites. Using the *brms* package [53], we fit a Bayesian multivariate mixed-effect model to explain the variability and covariance of two response variables describing the characteristics of the bedsite (bedsite visibility and its distance to the human feeding hotspot) as a function of the following predictors: begging rank and age of the mother; weight of the fawn at capture and its sex. An advantage of this modelling approach is that it tests for the effect of independent variables on one of the two response variables while taking the other response variable into account and vice versa, therefore avoiding fitting two less comprehensive univariate models. Our model *a priori* structure is as follows:


$$\left(Bedsite\;visibility\;+Distance\;to\;feeding\;hotspot\right)∼\;Mother^{\prime }s\;begging\;rank\;+Fawn\;weight+Mother^{\prime }s\;age+Sex+Mother^{\prime }s\;begging\;rank^{*}Fawn\;weight+Mother^{\prime }s\;age^{*}Fawn\;weight+Mother\;ID+Year$$

We included the interaction between mothers’ begging rank and fawn weight (proxy for age) to test our hypothesis that lighter (younger) fawns and related bedsite characteristics will be more strongly driven by mothers’ begging rank, as opposed to older fawns which can be more independent, mobile and less driven by mothers in terms of hiding. Our experience in the field across multiple fawning seasons suggested that older and heavier fawns tend to move more independently and they can select their own spot to rest. In comparison, younger and lighter fawns are usually observed where the mother left them, suggesting to us that the mother has a direct role in selecting the bedsites (primarily for younger fawns). Table 1 provides a summary of all the variables used. We also included the interaction between mother's age and fawn weight to control for the fact that more experienced (older) females may conceal their neonate fawns better. All numerical predictors were scaled (row value minus the sample mean eventually divided by the sample standard deviation) to improve model convergence. Including both single and quadratic terms of our explanatory variables resulted in increased uncertainty in the model and overfitting, similar ecological conclusions of the model were evident without quadratic terms. Therefore, based on the inspection of the model fit and residual patterns, we were satisfied by the fact that the inclusion of single terms and two-way interactions gave the model sufficient flexibility to account for nonlinear effects. Finally, the identity of the mother and year of capture were included in the model as crossed random intercepts to account for mothers who may have birthed multiple fawns within our study period. All predictors included in the model were successfully screened for collinearity issues (|*r*_p_| < 0.7) [54]. The begging rank of mothers was slightly correlated with their age but far from collinear, with a Pearson correlation coefficient, *r*_p_ = 0.11 (*t* = 1.85 and *p* = 0.06), showing the tendency of older mothers to be more likely to beg for food than younger ones (*sensu* Griffin *et al.* [7]). This allowed us to include both predictors in the *a priori* model and assess the effect of the begging rank of mothers on selected bedsite characteristics while accounting for age and experience. All data handling and analyses, including statistical and geographic information system (GIS) analyses, were carried out using R 4.0.5 [55].

**Table 1.** Summary of the variables used in the model. All numerical predictors were scaled (mean-centred and divided by sample standard deviation) to improve model convergence.

variable	description	scaled
bedsite visibility (BV)	visibility of bedsite to predators (ranging from 0 to 1)	yes
distance to feeding hotspot	distance to popular human feeding hotspot within the park (in m)	yes
mother’s begging rank	numerical score measuring how consistently mother accepts food from humans (ranging from −2.16 to 5.10)	yes
fawn weight	weight of fawn at capture (in kg)	yes
mother’s age	age of mother (proxy for experience, in years old)	yes
sex	sex of fawn (m/f)	no
mother ID	identity of the mother	no
year	year of fawn capture (four levels, 2018–2021)	no

## 3. Results

Data were collected from 281 capture events of 172 neonate fawns born to 110 mothers. The dataset encompasses multiple fawning seasons over 4 subsequent years (2018–2021 inclusive). Fawns (*n* = 172) were evenly distributed across the study with 42 fawns in 2018, 38 in 2019, 45 in 2020 and 47 in 2021. Ninety-one fawns were captured once, 57 twice, 20 three times, and only 4 were captured four times within the same fawning season. The age of mothers ranged between 2 and 17 yrs (mean: 6.7 yr) over the study period. Among the 110 mothers included in the study, 64 gave birth to one fawn only, 33 to two, 10 to three and only three females gave birth to four fawns over 4 yrs. These findings confirm the low productivity in the herd, with most females skipping one or more fawning seasons before giving birth to a fawn surviving the first three months after birth.

As reported in table 2, the parameters estimated by our Bayesian multivariate mixed-effect model explain the variability of the two response variables (bedsite visibility and its distance to the people feeding hotspot, both fitted with the Gaussian distribution of errors) inclusive of the variation of the crossed random intercepts (mother’s identity and year of study). The model explained 22% of the variability in bedsite visibility (Bayes *R* ^2^ = 0.22: 2.5% and 97.5 quantiles: 0.12–0.36), whereas it explained 58% of the variability of the distance to the hotspot (Bayes *R* ^2^ = 0.58: 2.5% and 97.5 quantiles: 0.50–0.64). We did not find a clear covariance between bedsite visibility and their distance to the people feeding hotspot (group-level effects in table 2), indicating that bedsites with low visibility could be found both close to and far from the feeding hotspot. In relation to population-level effects, specifically referring to single effects not included within interactions, we found no effect of sex of the fawn on the characteristics of the bedsite (both visibility and distance to the human feeding hotspot, table 2). In relation to the interaction terms specifically included to test our *a priori* hypotheses, when the distance to the human hotspot was the response variable, we found clear effects of the interactions of mother’s begging ranks with fawn’s weight (proxy for age), as well as the interaction between mother’s age (proxy for experience) and fawn’s weight (table 2) for both response variables. We have expanded these results below along with relevant figures.

**Table 2.** Parameters estimated using the Bayesian multivariate mixed-effect model explaining the covariation of the two response variables (bedsite visibility (BV) and its distance to the people feeding hotspot (DH), both fitted with the Gaussian distribution of errors) as a function of mother’s begging rank and age, weight and sex of the fawn inclusive of *a priori* interactive effects. Mother identity and year of study were both fitted as crossed random intercepts. The model was fitted on *n* = 281 observations drawing from three chains, each with 8000 iterations (warm-up = 500, thin = 2 and total post-warm-up draws = 11 250). To improve readability, asterisks (*) are added to indicate estimate and related 95% confidence intervals not passing zero.

	estimate	est. error	l-95% CI	U-95% CI	Rhat	bulk ESS	tail ESS
group-level effects							
mother							
intercept s.d. (BV)	0.21*	0.15	0.01	0.53	1	1600	4392
intercept s.d. (DH)	0.70*	0.07	0.57	0.84	1	4947	8437
intercept correlation (BV versus DH)	−0.11	0.40	−0.90	0.75	1	434	674
year							
intercept s.d. (BV)	0.53*	0.39	0.13	1.56	1	4591	6871
intercept s.d. (DH)	0.15*	0.17	0.00	0.61	1	4862	3218
intercept correlation (BV versus DH)	0.14	0.58	−0.92	0.97	1	8800	8379
population-level effects							
intercept (BV)	−0.01	0.31	−0.69	0.64	1	4671	5445
intercept (DH)	0.02	0.14	−0.25	0.30	1	4988	4687
mother’s begging rank (BV)	−0.08	0.06	−0.20	0.04	1	9421	9833
mother’s begging rank (DH)	0.10	0.06	−0.02	0.23	1	8052	7433
fawn’s weight (BV)	−0.30*	0.06	−0.42	−0.18	1	8740	9807
fawn’s weight (DH)	−0.10*	0.05	−0.20	0.00	1	8843	8680
mother’s age (BV)	−0.14*	0.07	−0.27	−0.01	1	7407	1 01 094
mother’s age (DH)	0.17*	0.08	0.01	0.33	1	7852	8670
sex of fawn (m) (BV)	−0.04	0.12	−0.28	0.20	1	8136	8714
sex of fawn (m) (DH)	0.05	0.11	−0.16	0.26	1	9558	10 001
mother’s begging rank × fawn’s weight (BV)	0.11	0.06	−0.01	0.23	1	8724	8144
mother’s begging rank × fawn’s weight (DH)	−0.13*	0.05	−0.23	−0.02	1	8747	9484
fawn’s weight × mother’s age (BV)	0.12*	0.05	0.01	0.22	1	8168	8604
fawn’s weight × mother’s age (DH)	−0.10*	0.05	−0.19	−0.00	1	9090	6734
family specific parameters							
sigma (BV)	0.90	0.05	0.80	1.00	1	3061	5788
sigma (DH)	0.66	0.04	0.59	0.73	1	6397	8885

*Notes:* BV, bedsite visibility is the response variable ; DH, distance to the human hotspot is the response variable; ESS, explained sum of squares.

**Figure 2.**
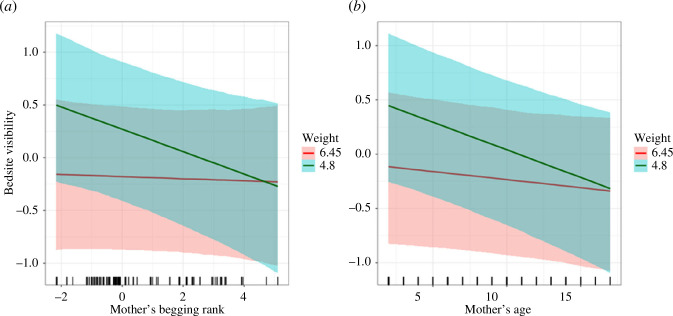
Effect of (*a*) begging rank of the mother (*x*-axis) and (*b*) mother’s age (years old, *x*-axis) interacted with the weight of the fawn (first and third quartile, in kg) on the visibility (scaled, *y*-axis) of bedsites as predicted by the Bayesian multivariate mixed-effect model where the error bands represent 95% credible intervals. Begging mothers (higher begging rank) as well as older and more experienced mothers tended to hide their fawns in less visible sites (lower visibility) when the fawns were younger (lighter weight), whereas such a relationship was absent in heavier (older) and more independent fawns.

**Figure 3.**
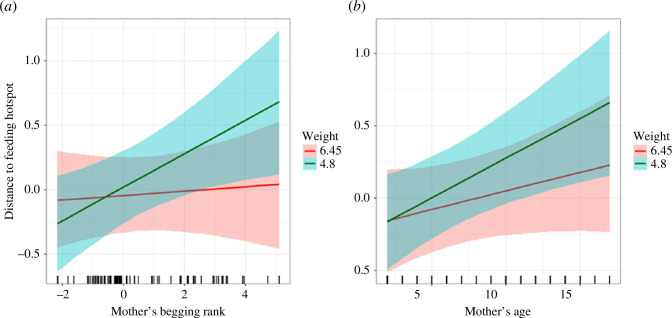
Effect of (*a*) begging rank of the mother (*x*-axis) and (*b*) mother’s age (years old, *x*-axis) interacted with the weight of the fawn (first and third quartile, in kg) on the distance to feeding hotspot (scaled, *y*-axis) of bedsites as predicted by the Bayesian multivariate mixed-effect model where the error bands represent 95% credible intervals. Fawns were hidden in sites further from feeding hotspots by both consistent beggar mothers and older and more experienced ones, whereas such a relationship was absent in heavier (older) and more independent fawns. Note that, in the plot, a scaled value of distance 1 corresponds to 901 m and a scaled distance of −0.5 corresponds to 476 m.

We found a strong tendency among consistent beggar mothers (i.e. higher begging rank) to conceal their fawns in sites with reduced visibility (figure 2*a*), but this was true for younger (lighter) fawns only and not for older (heavier) and more mobile fawns. Contrary to our main expectation, consistent beggar mother’s fawns were found in areas further away from the hotspot of human feeding when compared with the fawns of shyer mothers with lower begging ranks (figure 3*a*). Again, this pattern was visible only when the fawns were younger (lighter) and vanished for older and heavier fawns (figure 3*a*).

When looking at the interaction of mother’s age and fawn weights (table 2), accounting for the effect of the begging rank, older and more experienced mothers concealed their fawns in less visible sites (figure 2*b*) and more distant to the hotspot of human feeding (figure 3*b*). Similar to the interaction with mother’s begging rank, the pattern was evident in younger (lighter) fawns only and vanished in older and heavier fawns.

## 4. Discussion

Our aim was to disentangle the anti-predator strategies of mothers showing varying degrees of willingness to interact with humans in a peri-urban environment during the birth period. Bedsite selection is an incredibly important behavioural decision by mothers of hider species because fawn survival can depend on multiple factors including predation, disease and hypothermia [39,56]. Mothers can limit these threats by selecting suitable bedsite locations, especially where a fawn’s greatest defence from predation is cover, cryptic coloration [57] and adequate shelter from cold and damp weather conditions (which can help to fend off hypothermia and disease). Our main *a priori* expectation was that female deer that show a reduced fear response to humans and regularly accept food from them (*sensu* Griffin *et al.* [7]) would have concealed their fawns closer to the hotspot of artificial feeding. By doing so, they could more easily exploit artificial food while remaining relatively close to their concealed offspring, facilitating maternal care responsibilities such as suckling. Contrary to our expectation, consistent beggar females that showed the most adaptable behaviour compared with shyer females, in that they took advantage of artificial feeding opportunities [7], concealed their fawns in areas that tended to have thicker vegetation and were farther away from human feeding hotspots. Our work not only describes in detail inter-individual variability in coping with humans within the same population but also raises new questions which we expand upon further below. Our results clearly document a remarkable adaptation to local ecological conditions shown by a subset of female deer living in an urban park.

Our *a priori* hypothesis that the link between mothers’ willingness to accept food from humans and bedsite selection closer to human hotspots would be stronger during the first days of a fawn’s life—when the decision of where the neonate fawn will be delivered and concealed is expected to be entirely taken by the mother—and weaker for bedsite locations occupied by older, more mobile and independent fawns. Our data confirmed this prediction and we showed that the link between bedsite characteristics and mothers’ behaviour was evident only in captures of young neonates usually within a week after birth, meaning that the decision to choose this location was driven by the mother [58]. As fawns mature, mothers begin using contact calls to locate them, suggesting that they have only an approximate knowledge of the fawn’s location [40]. This suggests that fawns begin to take an active role in deciding their location and move around more independently of their mothers after the first period of the hiding phase. Additionally, the same bedsite preferences shown by begging females are adopted by older mothers. In general, older females would have gained experience from raising previous offspring and, therefore, females tend to become better mothers as they age [59]. However, consistent beggar mothers vary across the age range within our population with some being much younger and less experienced mothers than others. This illustrates that individuals engaging in begging activities have acquired beneficial behavioural traits normally associated with older, wiser and more experienced mothers.

Our study is potentially biased towards successful mothers and higher quality fawns, which makes our patterns even more striking considering that we are missing the lighter fawns of non-beggar mothers. Our mother–fawn pairs are limited to the fawns that survived at least a few weeks to be able to join the female herd and become available for our direct observations; therefore, we are missing the lower quality fawns that died very soon after birth or during the hiding phase (i.e. within four weeks after birth). In the same population, it has been shown that lower birthweight individuals die within the first few weeks after birth [48]. In the period of 2018–2021, each year, we tagged between 83 and 102 newborns [48] and collected data on mother–fawn pairs as well as mothers’ begging ranks on a subsample of fawns between 38 and 47 (this study). The difference in sample size between the fawns we monitored over the summer and those tagged at the beginning of it was due to (i) approximately 15% of the newborns being the offspring of a non-ear-tagged mother, therefore excluded, and (ii) approximately 15–20% of the fawns not surviving the first weeks of life [48]. We found a direct link between mothers’ begging behaviour and anti-predator strategies across a range of fawns varying from top quality to mid–low quality, and we would expect patterns to be even stronger by including the lower quality fawns. Genetic pedigree analysis could be the next step to fill this gap of knowledge that we are unable to tackle with our study.

It has previously been shown that bolder mothers (the same females included in this study) receive more food than their shy conspecifics, resulting in them giving birth to fawns that are typically 300–500 g heavier [7,32]. Not only are these fawns heavier but they also exhibit higher growth rates than the offspring of mothers who do not beg [32]. This provides their offspring with an advantage early in life when weight is an important predictor for neonate survival [48] and mortality is at its highest for ungulate species [60]. Considering that neonate fawns rely solely on their mother’s milk for nutrition in early life [45], this could be linked to findings that show lactating females who receive supplementary feeding have increased milk production [61]. Previous research has also shown that prolonged nursing occurs in heavier offspring in other ungulates [62]. Combining these early-life characteristics of higher birth weight and faster growth rates [7,32,48] with our findings about bedsite characteristics (low visibility) and location (more distant from humans and their dogs compared with the other females in this population) [34], the offspring of bolder mothers are consistently awarded multiple survival advantages (to be further explored by future research) from *in utero* to young adult life stages. This provides evidence that begging mothers are better adapted to this environment than shyer females.

While individuals who regularly engage in human feeding activities may be better adapted in this context, it is important to note that bolder behaviour is not always advantageous [29]; for instance, individual deer in the Phoenix Park that approach humans to get food might have higher mortality risks in a different ecological context and more natural settings (e.g. where hunting pressure would be greater and more likely to target approachable individuals). Also the link between the increased likelihood of offspring survival due to multiple factors, like increased birth weight, growth rates [7,32,48] and superior bedsite, as shown here, and food acceptance may be context dependent and artificial food could not be necessarily be linked to benefits for the acceptors. It has been argued that feeding activities should only be deemed acceptable, ‘if it could be controlled, has a beneficial conservation effect and does not compromise an animal’s long-term welfare’ [18]. Within our site, feeding can be opportunistic, whereby visitors offer food that was originally brought for their own consumption (or plants sourced from the park), or premeditated, whereby food items such as carrots have been brought with the intention of feeding the deer (figure 1). This can lead to the issue of deer in the park often being given foods that are vastly different from their natural diets (e.g. chocolate, crisps/chips and sandwiches). Regular ingestion of food unsuitable for their natural diet has led to physiological changes within the rumen of the deer, with consistent beggars showing increased papillae density [33]. When an animal is regularly exposed to human food that they cannot digest, it worsens their physical health leaving them more susceptible to parasites and diseases [63] that could be passed on to other healthy (potentially non-begging) members of the population.

While bold behaviour may be rewarded in some cases within an intraspecies context through fitness advantages over shyer conspecifics [7,32,48], it is questionable whether it is beneficial when we consider inter-species relations. In this landscape, humans and deer are constantly in close proximity due to the size and use of our study site. As previously mentioned, bold behavioural types have been linked to behaviours like aggression and risk-taking [18,19], which can cause injury (both to deer and people). These interactions can lead to increased human–wildlife conflicts, which in turn may require more robust management strategies (and financial investment) to monitor and minimize these problems.

Increased research into the adaptability of species to urban settings has led to concerns about the non-random sorting of individuals [5] with the urgency to improve our understanding of the mechanisms through which behaviour helps animals to cope with such environmental alterations. Previous studies have shown that extractive activities like hunting and fishing are pushing selection towards less desirable traits in wild animals [64]. Based on our bedsite selection findings coupled with previous findings of increased fawn birth weights and growth rates [7,32,48], consistent beggar mothers seem to have higher reproductive success than their conspecifics that beg less. It could be argued that feeding activities promote the artificial selection of bolder begging behaviours [7,12]. In a more natural setting, this behavioural type would exist within a herd, but the proportion of bold to shy individuals would be maintained through the associated costs of boldness, e.g. predation [65]. However, owing to the lack of natural predation for bolder adults in this circumstance, boldness can continually be rewarded with additional food without the same level of associated risks, which could therefore be encouraging this behaviour. If artificial selection is occurring and bolder mother’s offspring are surviving better than their shyer conspecifics, we could see an increase in the proportion of bold individuals in populations over time (yet to be investigated).

This research advances our understanding of human–wildlife interactions and related knock-on effects for offspring, but several questions remain unanswered. The next steps in better understanding the impacts of these close contact associations between humans and wildlife are to allocate research efforts to understand whether these behaviours are passed down through generations and the mechanisms involved. Griffin *et al.* [7] documented high intra-individual repeatability in fallow deer begging behaviour across years, suggesting that personality could play a role in driving the behaviour of the bold beggars. This would be particularly problematic if the innate propensity to interact with humans is a heritable trait like other personality dimensions (e.g. [66–68]). It is not yet understood whether this propensity would be inherited genetically or, alternatively or in addition, if cultural transmission of begging behaviours occurs from mother to offspring, either or both contributing to the maintenance and potential increase in the frequency of this behaviour over generations. Either way, it must be a research priority to better understand how humans are, voluntarily or not, shaping the behaviour of wildlife within increasingly human-dominated landscapes.

## Data Availability

The dataset generated and analysed during the study is available in the figshare repository [69]. We include all files necessary to run the analysis in R to ensure a completely transparent and open-science approach.
